# Seamless and orthogonal expression of genetic parts in polyhydroxyalkanoate (PHA)-producing bacterial chassis for plastic bio-upcycling applications

**DOI:** 10.1016/j.mex.2023.102434

**Published:** 2023-10-11

**Authors:** Matthlessa Matthew Minggu, Nur Anisza Hanoum Naseron, Hazlam Shamin Ahmad Shaberi, Nor Azlan Nor Muhammad, Syarul Nataqain Baharum, Ahmad Bazli Ramzi

**Affiliations:** Institute of Systems Biology (INBIOSIS), Universiti Kebangsaan Malaysia (UKM), 43600 UKM Bangi, Selangor, Malaysia

**Keywords:** pSV-Ortho-PHA (pSVOP) expression system, Orthogonal expression, PHA-producing bacteria, *Pseudomonas putida*, *Cupriavidus necato*r, Seamless cloning, Plastic bio-upcycling

## Abstract

Polyhydroxyalkanoate (PHA)-producing bacteria represent a powerful synthetic biology chassis for waste bioconversion and bio-upcycling where PHAs can be produced as the final products. In this study, we present a seamless plasmid construction for orthogonal expression of recombinant PET hydrolase (PETase) in model PHA-producing bacteria *P. putida* and *C. necator*. To this end, this study described seamless cloning and expression methods utilizing SureVector (SV) system for generating pSV-Ortho-PHA (pSVOP) expression platform in bioengineered *P. putida* and *C. necator*. Genetic parts specifically Trc promoter, pBBR1 origin of replication, anchoring proteins and signal sequences were utilized for the transformation of pSVOP-based plasmid in electrocompetent cells and orthogonal expression of PETase in both *P. putida* and *C. necator*. Validation steps in confirming functional expression of PETase activity in corresponding PETase-expressing strains were also described to demonstrate seamless and detailed methods in establishing bioengineered *P. putida* and *C. necator* as whole-cell biocatalysts tailored for plastic bio-upcycling.

• Seamless plasmid construction for orthogonal expression in PHA-producing bacteria.
• Step-by-step guide for high-efficiency generation of electrotransformants of *P. putida* and *C. necator.*
• Adaptable methods for rapid strain development (Design, Build, Test and Learn) for whole-cell biocatalysis.

## Graphical abstract

**Figure:**
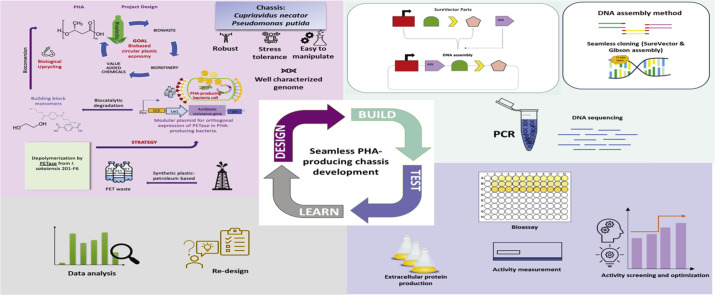

Specifications table

**Table:** 

Subject area:	*Biochemistry, Genetics and Molecular Biology*
More specific subject area:	Seamless strain design for plastic bio-upcycling
Name of your method:	pSV-Ortho-PHA (pSVOP) expression system
Name and reference of original method:	J.C. Braman, P.J. Sheffield, Seamless assembly of DNA parts into functional devices and higher order multi-device systems, PLoS One. 14 (2019) e0199653.
Resource availability:	All resources are commercially available

**Method details**

## Materials

The SureVector cloning system (Agilent Technologies, Santa Clara, USA) was employed in this study. For plasmid construction, the SureVector Core Kit (Agilent Technologies, Santa Clara, USA) was used according to the instruction manual. Oligonucleotides (Apical Scientific, Selangor, Malaysia) and gene fragments (Genewiz, New Jersey, USA) were chemically synthesized. Plasmid design was performed using SnapGene (SnapGene, Chicago, USA) plasmid design software.

## Bacterial strains, vectors, and growth conditions

*Escherichia coli* strains were routinely grown at 37 °C while *Pseudomonas putida* KT2440 (DSM 6125) and *Cupriavidus necator* H16 (DSM 428) were cultivated at 30 °C in Luria Bertani (LB) medium supplemented with antibiotic where appropriate. *Ideonella sakaiensis* 201-F6 (NBRC 110686) was grown in polypeptone media at 30 °C. All the bacterial strains, genes and plasmids used in this study were listed in Table 1.

**Table 1:** Strains, genes and plasmids used in this study.

Name	Description	Reference or source
Bacterial strains		
*E. coli XL1-Blue* Supercompetent Cells	recA1 endA1 gyrA96 thi-1 hsdR17 supE44 relA1 lac [F proAB lacIqZΔM15 Tn10 (Tetr)]	Agilent Technologies, USA
*E. coli* DH5α	General cloning host Δ(argF-lac)169, φ80dlacZ58(M15), ΔphoA8, glnX44(AS), deoR481, rfbC1, gyrA96(NalR), recA1, endA1, thiE1 and hsdR17	New England Biolabs, USA
*I. sakaiensis* 201-F6	Wild type	Biological Resource Center, NITE (NBRC), Japan
*P. putida* KT2440	mt-2 KT2440; Wild type	Leibniz-Institute DSMZ-German Collection of Microorganisms and Cell Cultures, Braunschweig (DSMZ), Germany
*C. necator* H16	Wild type	DSMZ, Germany
*E. coli XL1-Blue Supercompetent Cells*/pSV-INP+PETase+CBM	XL1-Blue containing pSV-INP+PETase+CBM, Km^R^	This work
*E. coli* DH5α/ pSVOP-INP+PETase+CBM	*E. coli* DH5α containing pSVOP-INP+PETase+CBM, Km^R^	This work
*E. coli* DH5α/ pSVOP-INP+PETase	*E. coli* DH5α containing pSVOP-INP+PETase, Km^R^	This work
*E. coli* DH5α/ pSVOP-ss-PETase	*E. coli* DH5α containing pSVOP-ss-PETase, Km^R^	This work
*E. coli* DH5α/ pSVOP	*E. coli* DH5α containing pSVOP, Km^R^	This work
*P. putida* KT2440/ pSVOP-INP-PETase-CBM	*P. putida* KT2440 containing pSVOP-INP+PETase+CBM, Km^R^	This work
*P. putida* KT2440/ pSVOP- INP-PETase	*P. putida* KT2440 containing pSVOP-INP+PETase, Km^R^	This work
*P. putida* KT2440/ pSVOP-ss-PETase	*P. putida* KT2440 containing pSVOP-ss-PETase, Km^R^	This work
*P. putida* KT2440/ pSVOP	*P. putida* KT2440 containing pSVOP, Km^R^	This work
*C. necator* H16/ pSVOP-INP-PETase-CBM	*C. necator* H16 containing pSVOP- INP+PETase+CBM, Km^R^	This work
*C. necator* H16/ pSVOP- INP-PETase	*C. necator* H16 containing pSVOP- INP+PETase, Km^R^	This work
*C. necator* H16/ pSVOP- ss-PETase	*C. necator* H16 containing pSVOP-ss-PETase, Km^R^	This work
*C. necator* H16/ pSVOP	*C. necator* H16 containing pSVOP, Km^R^	This work
**Genes**		
PETase	PETase PCR amplified from *I. sakaiensis* 201-F6 (GenBank GAP38373.1)	This work
Promoter Trc (pTrc)	Strong *E. coli* promoter, a hybrid between the trp and lac UV5 promoters and synthesized as gene fragment	Genewiz, USA
Ice Nucleation Protein (INP)	Derived from *P. syringae* (GenBank accession number AF013159.1) and synthesized as gene fragment	Genewiz, USA
Carbohydrate Binding Module (CBM)	Derived from *P. stutzeri* (GenBank accession number AB012225.1) and synthesized as gene fragment	Genewiz, USA
pBBR1	pBBR1 replicon PCR amplified from pBBR1MCS-3 plasmid	This work
**Vector**		
pBBR1MCS-3	Source of the pBBR1 origin of replication, a broad host range vector with sized 5228 bp, Tet^R^	Nova Lifetech, China
**Recombinant vectors**		
pSV-INP+PETase+CBM	Recombinant vector constructed containing INP, PETase and CBM with pUC ori under the control of pTrc, Km^R^	This work
pSVOP-INP+PETase+CBM	Recombinant vector constructed containing INP, PETase and CBM with pBBR1 ori under the regulation of pTrc, Km^R^	This work
pSVOP-INP+PETase	Recombinant vector constructed containing INP and PETase with pBBR1 ori under the regulation of pTrc, Km^R^	This work
pSVOP-ss-PETase	Recombinant vector constructed containing PETase with its native signal sequence coming from *I. sakaiensis* 201-F6 with pBBR1 ori under the regulation of pTrc, Km^R^	This work
pSVOP	Recombinant vector constructed as backbone and control vector with pBBR1 ori under the regulation of pTrc, Km^R^	This work

### DNA preparation and manipulations

High-fidelity KOD FX NEO (Toyobo, Japan) was used for PCR amplification of DNA fragments used for construction and verification of the DNA fragments in recombinant vectors. All recombinant vectors were purified using FavorPrep™ Plasmid DNA Extraction Mini Kit (Favorgen, Taiwan). DNA fragments were purified from agarose gels using the NucleoSpin gel and PCR clean-up kit (Macherey-Nagel, Düren, Germany). The genomic DNA of *I. sakaiensis* was extracted using Wizard® Genomic DNA Purification Kit (Promega, USA) according to the manufacturer's instruction manual. DNA extractions and transformations in *E. coli* were performed according to standard molecular biology procedures [1]. All oligonucleotides used in this study were listed in Table 2.

**Table 2:** Oligonucleotides used in this study.

No.	Primer pairs	Sequence (5′−3′)	Description
1	Fwd PET	GACTGGATTGAAGTGAAGACTAGTCAGACCAA CCCCTACGCCCGCGG	Amplification of DNA fragment encoded PETase gene coming from *I. sakaiensis* 201-F6
	Rev PET	GCTGCAGTTCGCGGTGCGGAAGTCCGACACGC	
2	Rev pSV	**CTCGAGGAGATATTGTACACTAAACCAAATGG**	Amplification of DNA fragment encoded pTrc from synthesized gene fragment. The bold sequence is overlapping sequences for gene-of-interest PCR primers for vectors with a promoter module and an N-terminal tag module
	INP R SpeI	ACTAGTCTTCACTTCAATCCAGTCGTCGTCT	Amplification of DNA fragment encoded INP from synthesized gene fragment
3	Fwd CBD	GGACTTCCGCACCGCGAACTGCAGCAA	Amplification of DNA fragment encoded CBM from synthesized gene DNA fragment
	Fwd pSV CHI	**GGTGGCGGAGGTTCTGGAGGCG**	Amplification of CBM from synthesized gene fragment. The bold sequence is overlapping sequences for gene-of-interest PCR primers for vectors with a promoter module and an N-terminal tag module
4	Fwd pBBR1 NcoI	AAGGGGTTATGCTAGTCCATGGCTACCGGC GCGGCAGCGTTACCCGT	Amplification of DNA fragment encoding pBBR1 ori for construction of recombinant vector pSVOP-INP+PETase+CBM (NcoI site underlined)
	Rvr pBBR1 Nde I	GAAGGAGATATACATATGTCCCCCTACGG GCTTGCTCTCCGG	Amplification of DNA fragment encoding pBBR1 ori from vector pBBR1MCS-3 for construction of recombinant vector pSVOP-INP+PETase+CBM (NdeI site underlined)
5	Fwd pBBR1 KpnI SpeI	ATTTCACACAGGAAAGGTACCACTAGTCTACC GGCGCGGCAGCGTTACCC	Amplification of DNA fragment encoding pBBR1 ori from vector pBBR1MCS-3 for the construction of pSVOP (KpnI site underlined)
	Rvr pBBR1 Nde I	GAAGGAGATATACATATGTCCCCC TACGGGCTTGCTCTCCGG	Amplification of DNA fragment encoding pBBR1 ori from vector pBBR1MCS-3 for the construction of pSVOP (NdeI site underlined)
6	Fwd INP PETase KpnI	TTTCACACAGGAAAGGTACCATGA CGCTCGACAAAGCACTGGTGC	Amplification of DNA fragment encoding INP from vector pSV-INP+PETase+CBM (KpnI site underlined)
	Rev INP PETase EcoRI	GGCTGCCTCTAGAAGAATTCA GCTGCAGTTCGCGGTGCGGAAGTC	Amplification of DNA fragment encoding INP-PETase from vector pSV-INP+PETase+CBM (EcoRI site underlined)
7	Fwd ssPETase KpnI	CAATTTCACACAGGAAAGGTACCATGAA CTTTCCCCGCGCTTCCCGCC	Amplification of DNA fragment encoding PETase containing its native signal sequence from *I. sakaiensis* 201-F6 (KpnI site underlined)
	Rev ssPETase EcoRI	GGCTGCCTCTAGAAGAATTCTCAGCTG CAGTTCGCGGTGCGGAAG-3′	Amplification of DNA fragment encoding PETase containing its native signal sequence from *I. sakaiensis* 201-F6 (EcoRI site underlined)

### Genetic parts assembly using SureVector system

The SureVector system utilized a multipartite plasmid that provided seamless assembly of standard genetic parts for functional expression of genes in prokaryotic and eukaryotic hosts [2,3]. This modular cloning system provided streamlined and customizable DNA modules consisted of standard genetic parts for promoter (P), protein expression tag, selectable marker (SM), bacterial replication fragment (ORI), linkers (XP) and repressor proteins that were designed to be flanked by unique 30 base pair (bp) sequences. The SureVector platform allowed user-defined plasmid construction via the introduction of DNA sequences in the gene of interest (GOI) module.

### pSV-Ortho-PHA (pSVOP) plasmid design

**Fig. 1:**
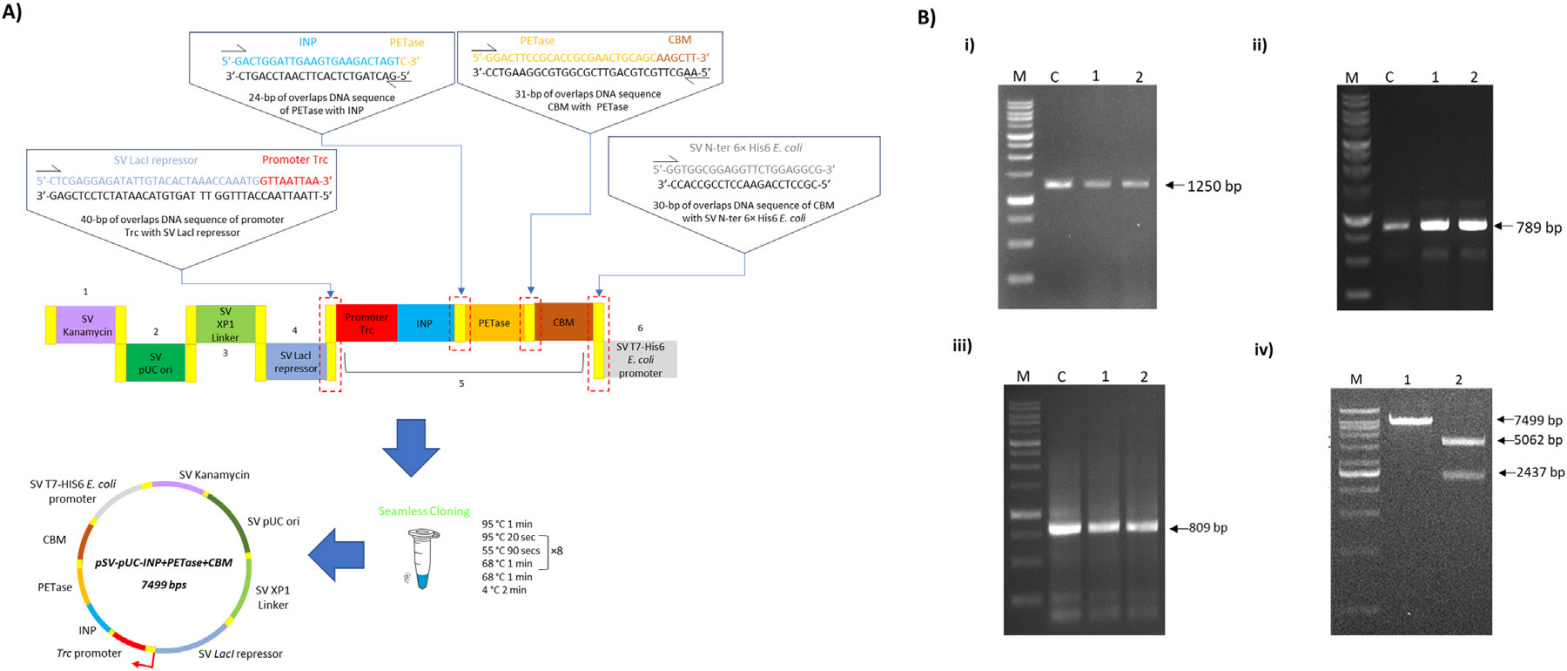
(A) The schematic illustrations of DNA assembly of SV parts and GOIs to produce pSV -INP+PETase+CBM, 7499 bp. (B) Validation of GOI and parts via colony PCR. Lane M: ExactMark 1 kb DNA Ladder, Lane C: Template DNA, Lane 1–2: Corresponding amplified genetic parts. (i) pTrc + INP, 1250 bp (ii) PETase, 789 bp (iii) CBM, 809 bp. (iv) Double digestion product of pSV -INP+PETase+CBM, 7499 bp using PacI and EcoRI. Lane M: VC 1 kb DNA Ladder; Lane 1: Undigested plasmid; Lane 2: Plasmid digested with PacI and EcoRI.

As a proof-of-concept for orthogonal expression, PETase (Genbank GAP38373.1) gene from *I. sakaiensis* was selected as the main genetic part for plastic biocatalysis in two different PHA-producing bacteria specifically *P. putida* and *C. necator*. To enable plasmid replication and functional expression in both bacterial strains, pBBR1 origin of replication (ori) and Trc promoter (pTrc) were selected as parts for conferring orthogonal expression in both bacteria. To demonstrate the feasibility of extracellular production of PETase, ice nucleation protein (INP; *P. syringae* Genbank AF013159.1), carbohydrate binding domain (CBM; *P. stutzeri* Genbank AB012225.1) and signal sequence (SS; *I. sakaiensis* Genbank GAP38373.1) sequences were added to generate pSVOP-INP-PETase, pSVOP-INP-PETase-CBM and pSVOP-ss-PETase plasmids, respectively. The design and construction of pSVOP plasmid harboring the corresponding genetic SV parts and GOIs were illustrated in Fig. 1.

### Parts assembly for generating recombinant plasmids

**Fig. 2:**
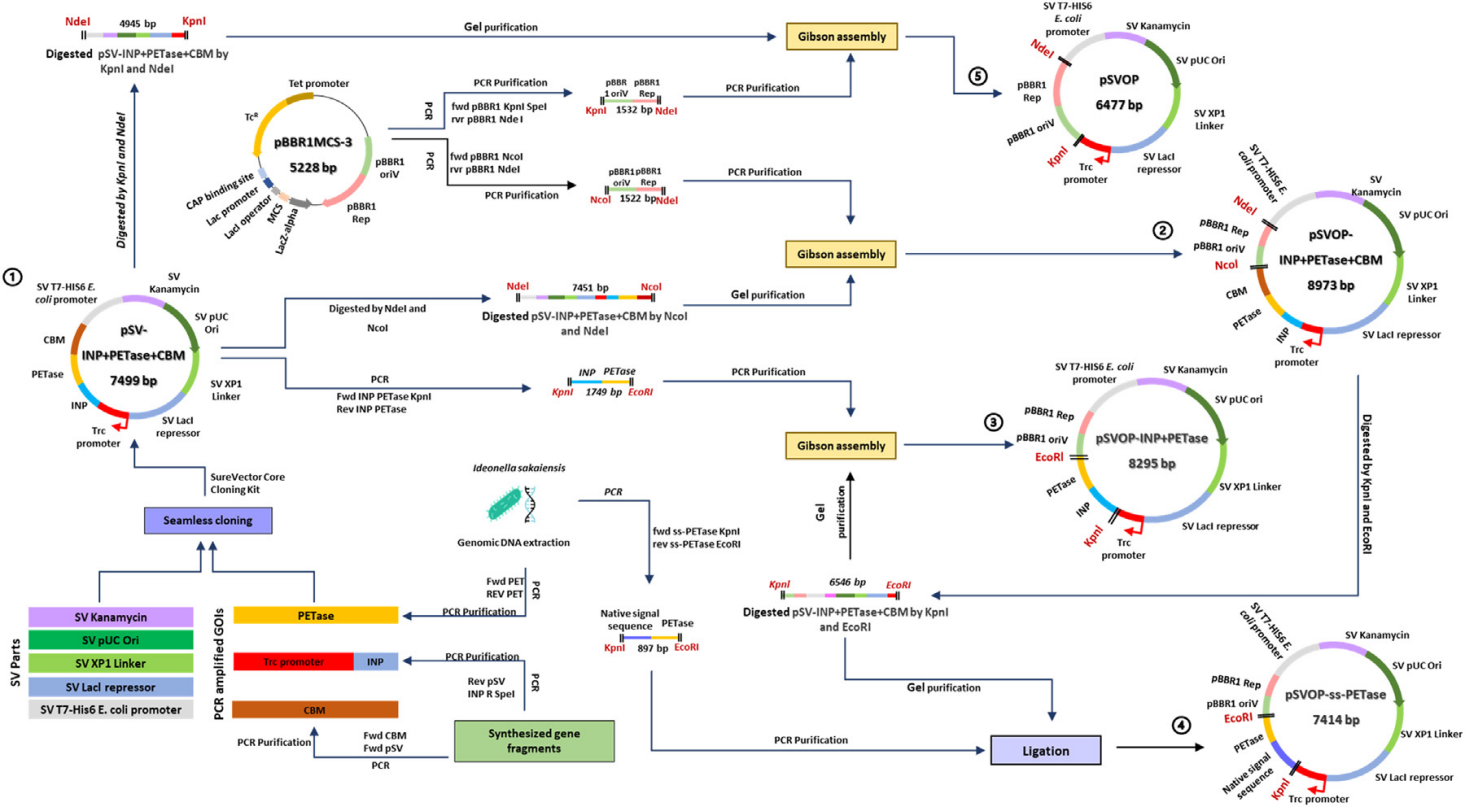
Graphical overview of overall plasmid design and construction using seamless SureVector platform and Gibson assembly.

Using the SureVector Core cloning kit, all the genetic parts were introduced in the GOI module to generate the starting plasmid, pSV-INP+PETase+CBM (Fig. 1). The genetic parts were assembled according to the gene design in the manufacturer's instruction manual. All the modules were assembled as follows in a final volume of 20 μL: 2 µL of 10× SV reaction buffer, 2 µL of 1× SureSolution, 1 µL of dNTP Mix, 1 µL of SV Enzyme, 2 µL of each module consisting SM kanamycin, ORI pUC, XP-1 XP1 Linker, XP-2 Lacl and P T7-His6, 0.05 p/mol µL of GOI fragments consisting DNA fragments of promoter pTrc, INP, PETase, CBM and pBBR1. Thermocycling of these components consisted of 1 cycle of 95 °C for 1 min, followed by 8 cycles at 95 °C for 20 s, 55 °C for 90 s and 68 °C for 1 min; 1 cycle at 68 °C for 1 min, and 1 cycle at 4 °C for 2 min. Following thermocycling, one unit of DpnI restriction enzyme was added to the reaction and incubated for 5 min at 37°C followed by transfer on ice. One μL of the reaction was transformed into *E. coli* XL1-Blue Supercompetent cells according to manufacturer's instructions. The transformation mixtures were spread onto LB agar plates containing the appropriate antibiotic and incubated at 37°C until colonies were easily visualized (12–16 h). The assembled pSV-INP+PETase+CBM plasmid was validated via Sanger sequencing (Apical Scientific, Selangor, Malaysia). To generate pSVOP plasmids, broad-host-range pBBR1 ori from pBBR1MCS-3 plasmid (Nova Lifetech Inc., Hong Kong, China) was used and introduced to generate corresponding plasmids, pSVOP, pSVOP-INP-PETase-CBM, pSVOP-INP-PETase and pSVOP-ssPETase (Fig. 2). The respective plasmids were assembled in the shown numbering order via isothermal-based Gibson assembly using methods described previously by Ramzi, A.B., et al. (2018) [4].

### Transformation of recombinant PETase expression vectors systems in PHB-producing bacteria by electroporation

#### Preparation of electrocompetent cells and electroporation of *P. putida*

**Fig. 3:**
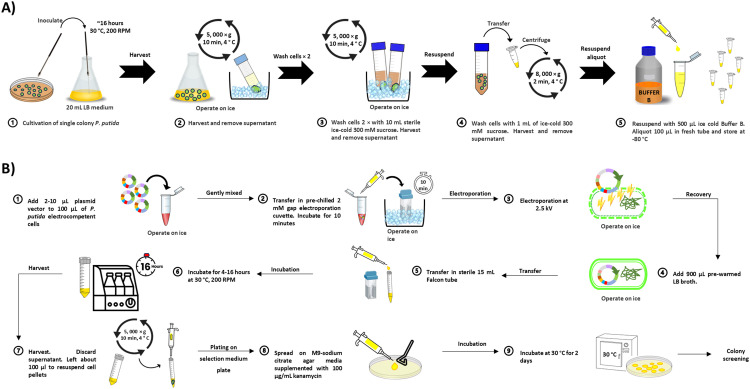
The schematic illustrations of (A) preparation of electrocompetent cells and (B) electroporation of plasmids into *P. putida* KT2440 electrocompetent cells.

The preparation of *P. putida* electrocompetent cells and electroporation methods were adapted from Martínez-García, E. and deLorenzo,V. (2012) [5]. A graphical overview of the protocols for preparing and transforming the recombinant plasmids into electrocompetent *P. putida* was shown in Fig 3. A single colony from a freshly streaked plate of *P. putida* was inoculated in 20 mL LB medium as starter culture and grown overnight (∼16 h) at 30 °C with shaking (200 RPM). The culture was immediately placed on ice and cells were harvested by centrifugation at 5, 000 × g at room temperature for 10 min. The cells were washed twice with sterile ice-cold 300 mM sucrose with centrifugation at low temperatures. Next, the harvested cells were gently resuspended in 1 mL of ice-cold 300 mM sucrose followed by transferred into sterile 1.5 mL Eppendorf tubes. The cell culture tubes were centrifuged at 8, 000 × g for 2 min. The supernatants were discarded. About 500 µL of ice-cold Buffer B (300 mM sucrose + 10 % (v/v) glycerol) was added to the cells and gently resuspended. The resuspended cells were distributed in 100 µL aliquots in sterile 1.5 mL Eppendorf tubes and stored at −80 °C (Fig. 3A). For electroporation (Fig. 3B), freshly prepared recombinant plasmids (Concentration range: 100 to 200 ng/µL; volume 2–10 µL) were added to 100 µL aliquot of *P. putida* competent cells and mixed gently. The samples were placed in a prechilled sterile 2-mm gap width electroporation cuvette (Bio-Rad, USA). The cuvettes were kept on ice for 10 min and then placed in the electroporation apparatus (Eppendorf Eporator®, Eppendorf AG, Germany) where the electrical settings voltage was set at 2500 V. Immediately after pulse discharge, 900 µL of pre-warmed LB medium was added directly to the cuvettes. The samples were transferred to 15-mL sterile test tubes and incubated at 30 °C overnight (∼16 h) with shaking (180 RPM). The cells were plated onto pre-warmed M9-citrate agar containing 100 µg/mL kanamycin. All plates were incubated for 2 days at 30 °C.

#### Preparation of electrocompetent cells and electroporation of *C. necator*

**Fig. 4:**
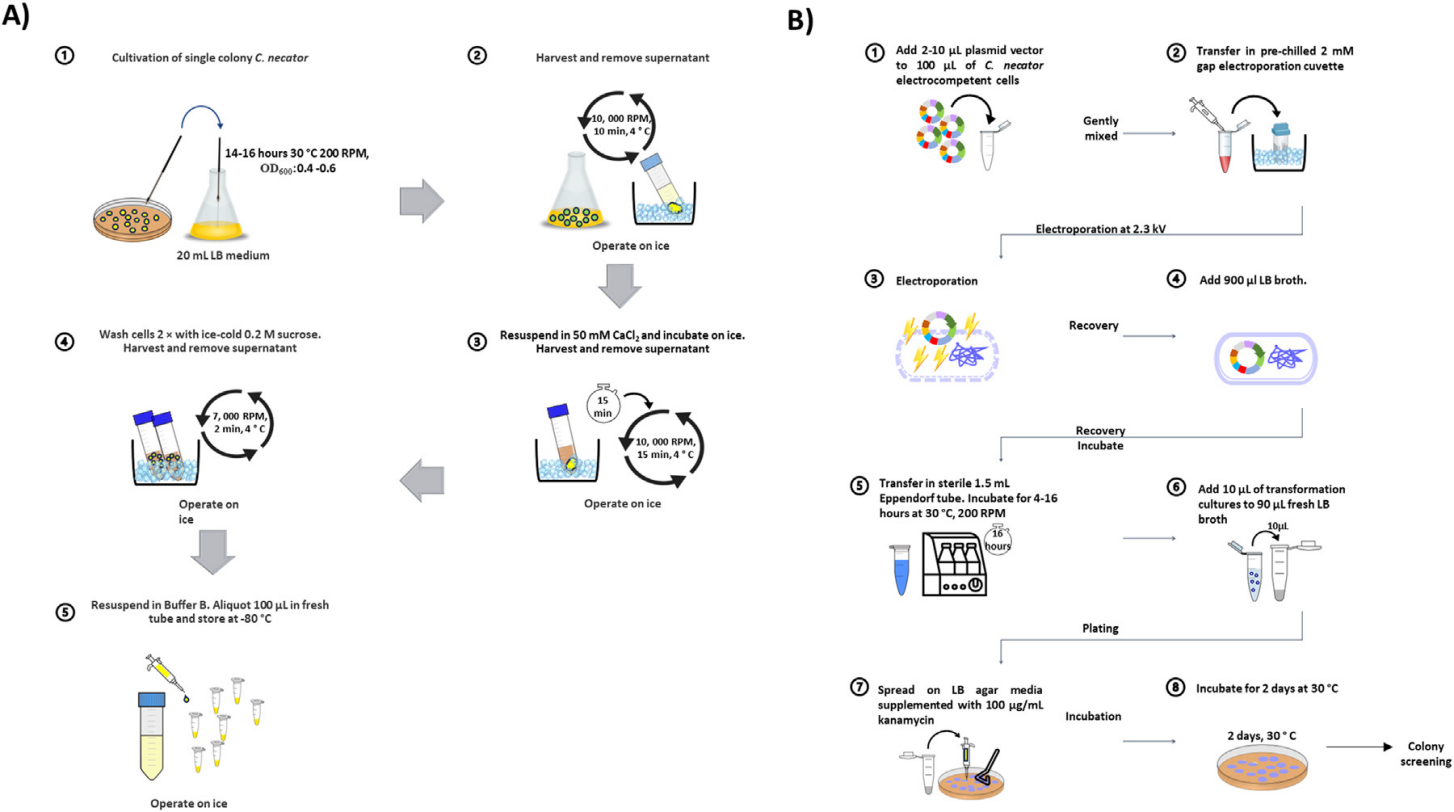
The schematic illustrations of (A) preparation of electrocompetent cells and (B) electroporation of plasmids into *C. necator* H16 electrocompetent cells.

The preparation of electrocompetent cells of *C. necator* and subsequent electroporation methods were modified from Tee, K. L., et al., (2017) [6]. A schematic overview of the protocols for preparing and transforming the recombinant plasmids into electrocompetent *C. necator* was illustrated in Fig. 4. As shown in Fig. 4A, a single colony of *C. necator* was cultured in fresh LB medium broth and incubated overnight at 30 °C with shaking (200 RPM). Preferably, the bacteria were grown until OD_600_ values reached 0.4 to 0.6. Following cultivation, the bacterial cells were harvested by centrifugation at 10,000 RPM for 15 min. The separated cells were resuspended in 50 mM CaCl_2_ and incubated on ice for 15 min. Cells were harvested following centrifugation for 10 min at 4 °C. Following this, the bacterial cells were then washed twice with ice-cold 0.2 M sucrose and centrifuged at 7000 RPM for 2 min 4 °C. All supernatants were removed completely. The washed cells were then resuspended in Buffer B (300 mM sucrose + 10 % (v/v) glycerol) and aliquoted into sterile 1.5 mL tubes (100 µL each). For electroporation steps (Fig. 4B), the competent cells of *C. necator* were mixed with freshly prepared recombinant plasmids (Concentration range: 100 to 200 ng/µL; volume 2–10 µL) in prechilled sterile 2-mm gap width electroporation cuvette (Bio-Rad, USA). The cuvettes were kept on ice for 10 min and then placed in the electroporation apparatus (Eppendorf Eporator®, Eppendorf AG, Germany) where the electrical settings voltage was set at 2300 V. Following pulse discharge, prewarmed LB broth was immediately added to the tube to make a total of 1 mL mixture to recover the cells. The bacterial cultures were then incubated at 30 °C 200 RPM overnight (∼ 18 h). The cultures were subsequently plated on LB+Kanamycin (100 µg/mL) plates. Plates were incubated for 2 days at 30 °C until colonies were formed.

## Method validation

### Colony PCR of PETase gene in PETase-expressing *P. putida* and *C. necator*

**Fig. 5:**
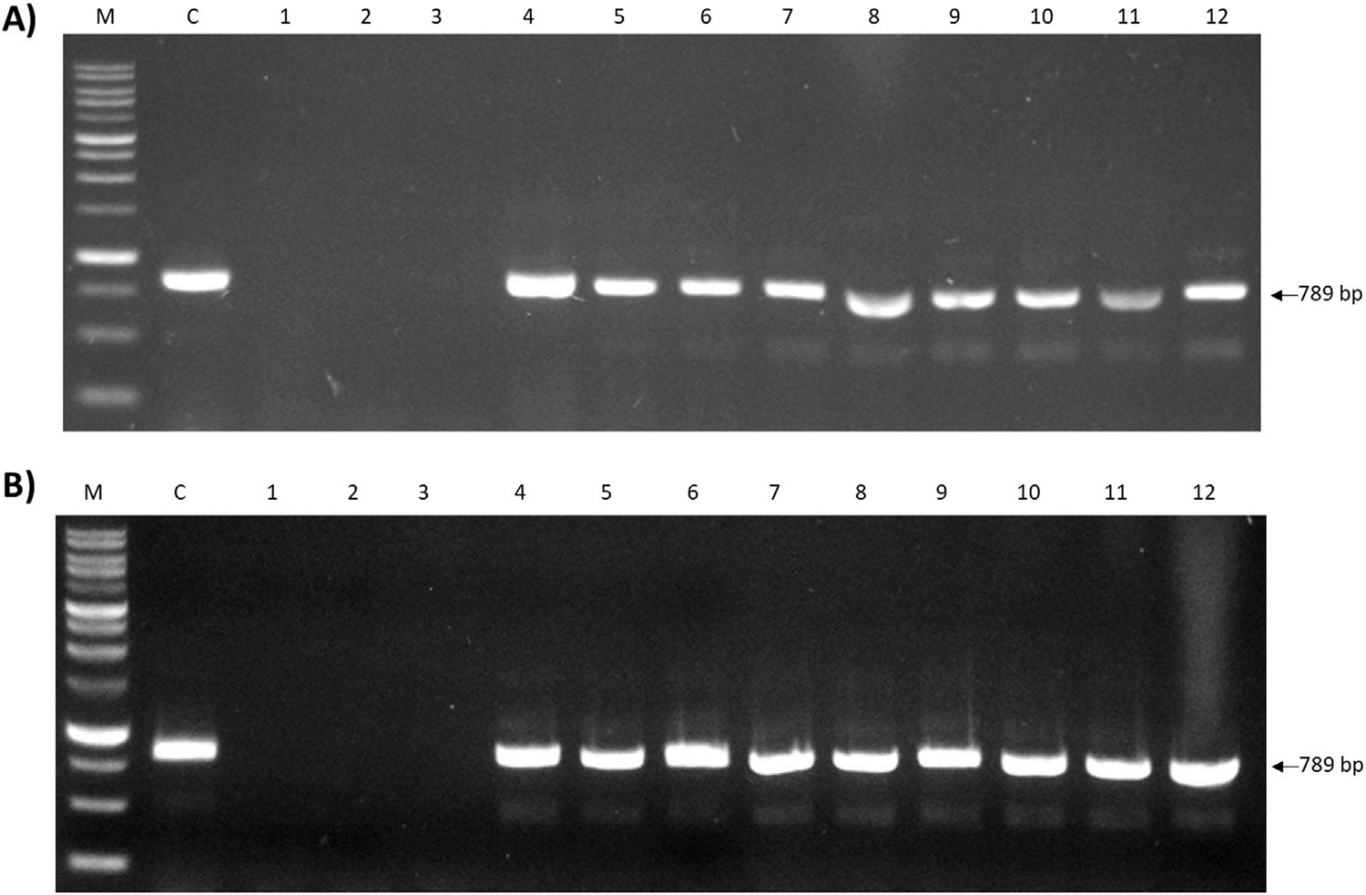
Validation of heterologously-expressed PETase gene in recombinant *P. putida* and *C. necator* transformed with corresponding plasmids. A) Colony PCR of recombinant *P. putida* transformants. Lane M: 1st Base 1 kb DNA ladder; Lane C: PETase gene from *I. sakaiensis* (Genomic DNA); Lane 1–3: pSVOP colonies; Lane 4–6: pSVOP-INP-PETase colonies; Lane 7–9: pSVOP-INP-PETase-CBM colonies; Lane 10–12: pSVOP-ss-PETase colonies. B) Colony PCR of recombinant *C. necator* transformants. Lane M:1st base 1 kb DNA ladder; Lane C: PETase gene from *I. sakaiensis* (Genomic DNA); Lane 1–3: pSVOP colonies; Lane 4–6: pSVOP-INP-PETase colonies; Lane 7–9: pSVOP-INP-PETase-CBM colonies; Lane 10–12: pSVOP-ss-PETase colonies.

To confirm the presence of recombinant plasmids in the transformants of *P. putida* and *C. necator*, colony PCR of recombinant PETase gene (789 bp) was performed by selecting random colonies on the plates of the corresponding recombinant strains. Each 25 µL of PCR mixture contained 4.5 µL autoclave distilled water, 12.5 µL 2× PCR buffer, 5 µL 2 mM DNTPs, 0.75 µL of 10 pmol of primer pairs of Fwd PET and Rev PET, 2 µL of template genomic DNA/transformant cells (Grown in LB+Kanamycin broth) and 0.5 µL of Toyobo KOD FX Neo (1 U/L). Thermocycling condition was set at; 1 cycle of pre-denaturation 94 °C for 2 min, 30 cycles of denaturation 98 °C for 10 s, annealing 71 °C for 30 s, extension 68 °C for 30 s. 1 cycle of final extension 68 °C and 4 °C. As shown in Fig. 5, single bands of PETase gene were obtained in all PETase-containing plasmid constructs hence confirming the high efficiency and successful introduction of the gene and respective plasmids in both bacterial hosts.

### Recombinant production of PETase protein in PHB-producing bacteria

**Fig. 6:**
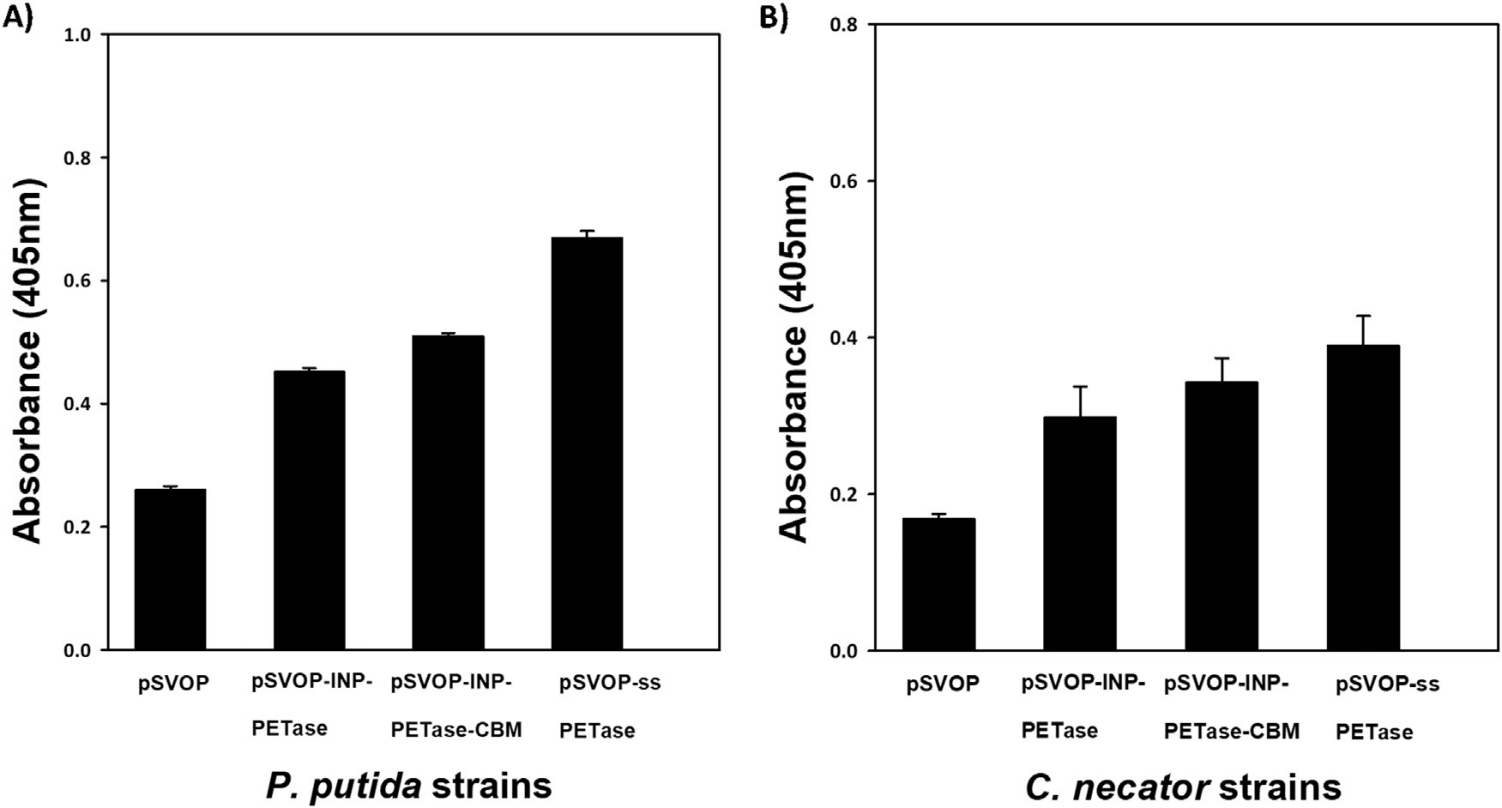
Functional expression of recombinant PETase in engineered *P. putida* and *C. necator* strains based enzymatic assays using PNPB as substrates. Strains containing ssPETase genetic part gave out the highest values in both *P. putid*a and *C. necator* strains (pSVOP strains were used as control negative strains). Values and error bars represent the mean and standard error of at least triplicate measurements.

Enzyme production was started by inoculation of an overnight culture of recombinant *P. putida* and *C. necator* harboring PETase expression vectors in LB plus kanamycin medium. The bacterial cultures were then induced using 0.01 M IPTG when the cell density reached approximately OD_600_= 0.4. The induced culture was harvested after 24 h-post induction and centrifuged 5000 × g*,*20 min at 4 °C. To demonstrate the functional activity of recombinant PETase in corresponding pSVOP plasmids, enzymatic assays were performed using para-nitrophenyl butyrate (PNPB) as the model esterase substrate using methods adapted from Heyde, S. H. A., et al., (2021) [7]. The esterase activity of the expressed PETase was measured spectrophotometrically in flat bottom polystyrene 96-well microtiter plates by measuring the absorbance reading at 405 nm using microplate absorbance reader (iMark™, Bio-Rad, USA). For this purpose, a 10 mM stock solution of PNPB was prepared by dissolving 13.9 mg of PNPB powder (Sigma Aldrich, USA) in 10 mL of acetonitrile. The assay mixture was composed of 100 µL of substrate-buffer solution (Tris–HCl pH 7.4 containing 2 mM of PNPB). The reaction was started by adding 50 µL of the bacterial supernatant with final OD_600_ = 0.1 (Diluted with LB broth) followed by incubation at 37 °C for 10 min. Activity measurements were performed in triplicate (Fig. 6).

## Conclusion

In line with the emerging synthetic biology strategies in utilizing PHA-producing bacteria for whole-cell biocatalysis, this study demonstrated detailed methods that enabled efficient generation and orthogonal expression of recombinant proteins in model PHA producers specifically *P. putida* and *C. necator*. The methods work efficiently for seamless and customizable strain design and can be adapted for strain optimization via the Design, Build, Test and Learn iterative synthetic biology cycle.

## CRediT author statement

Matthlessa Matthew Minggu: Conceptualization, Methodology, Validity tests, Writing, Reviewing, Editing. Nur Anisza Hanoum Naseron: Methodology, Validity tests. Hazlam Shamin Ahmad Shaberi: Data curation, Validity tests. Nor Azlan Nor Muhammad: Supervision, Reviewing. Syarul Nataqain Baharum: Supervision, Reviewing. Ahmad Bazli Ramzi: Supervision, Conceptualization, Writing, Reviewing, Editing.

## Declaration of Competing Interest

The authors declare that they have no known competing financial interests or personal relationships that could have appeared to influence the work reported in this paper.

## Data Availability

No data was used for the research described in the article.
